# Utilising spectral lighting simulation technique for evaluating transmitted daylight through glazing: Exploring the non-visual effects and colour appearance

**DOI:** 10.1016/j.heliyon.2023.e20436

**Published:** 2023-09-26

**Authors:** Marzieh Nazari, Barbara Matusiak, Oliver Stefani

**Affiliations:** a Light and Colour Centre, Department of Architecture and Technology, Faculty of Architecture and Design, Norwegian University of Science and Technology (NTNU), 7034, Trondheim, Norway; b Centre for Chronobiology, Psychiatric Hospital of the University of Basel, 4002, Basel, Switzerland; c Transfaculty Research Platform Molecular and Cognitive Neurosciences (MCN), University of Basel, 4002, Basel, Switzerland

**Keywords:** Spectral lighting simulation, Non-visual effects, Colour appearance, Smart glazing, Clear glazing, α-opic EDI

## Abstract

Modern humans spend more time indoors than their ancestors. In indoor environments, windows are the primary building elements that provide access to daylight and views. The advancement of the building industry has introduced new glazing and coating technologies for windows. Electrochromic glazing, in particular, has gained popularity in recent decades. These glazings’ tint varies with light exposure and electrical voltage, affecting the spectral power distribution of transmitted daylight. The growing knowledge of the impacts of light on sleep and health encourages an evaluation of the non-visual effects of daylight transmitted through glazing. Therefore, the aim of this paper is to investigate the non-visual effects of transmitted daylight through one clear and one smart glazing and evaluate the colour appearance variations. However, conventional visualisation tools are inadequate for this purpose, necessitating the use of alternative techniques that consider the spectral power distribution of light. To accomplish this, the Radiance-based Lark spectral lighting simulation tool was utilised. The non-visual effects were analysed by examining the responses of the five photoreceptors (Short-, Medium-, Long-wavelength cones, Rods, and ipRGCs) to light using the CIE spectral sensitivity functions. Additionally, the changes in colour appearance were assessed using six attributes: lightness, hue, chroma, vividness, depth, and clarity. The results demonstrate the effect of the studied glazing on non-visual light stimulation and colour appearance while presenting the challenges, applicability, and limitations of spectral simulation techniques. The proposed method yields promising results and can be a valuable tool for evaluating the effects of glazing on humans.

## 1 Introduction

Windows play a significant role in building design, offering both physical and psychological benefits to occupants. They provide access to daylight with its visual and non-visual effects, energy (solar gain), view (connecting to the outdoors), and enhance well-being [1]. Daylight delivered through the building envelope, such as windows, is a critical environmental factor that can influence the human perception of spaces [[2], [3], [4], [5]], as well as the productivity, satisfaction, and health of residents in the built environment [6,7]. Furthermore, daylight adds value to commercial office spaces, which highlights the need for its inclusion in design, policy, planning, and project funding [8].

For many years, the primary strategy to achieve visual comfort, energy savings, and prevention of overheating has been to limit sunlight in architectural spaces [9,10]. In theory, optimal thermal and visual comfort can be achieved through operable sun-shading systems, such as electrochromic or thermochromic glazings. In the past two decades, electrochromic glazing for windows has gained popularity due to its ease of control compared to blinds and louvres. The tint of these glazings changes when they are exposed to light and/or voltage changes. This ranges from maximum colour saturation of tint when being exposed to high levels of daylight (i.e., sunny sky conditions) to a bleach state, with minimum coloration, in low levels of daylight exposure (i.e., overcast sky conditions). Although the first generations of electrochromic glazing had a strong blue tint, current technology is developing new coatings and glazing that better maintain colour appearance while providing protection against solar radiation. *ChromoGenics Dynamic* is an example of the new generation of electrochromic glazing being studied in this research. This glazing has a brown tint and can be controlled by the supplied voltage; additionally, it automatically adjusts light transmittance in response to temperature fluctuations (solar radiation).

Tinted glazing has been the subject of several research projects focusing on glare, visual comfort, and energy consumption. However, as our understanding of the effects of light on circadian rhythms and health continues to evolve, the non-visual aspects of this type of glazing require special attention. Studies on the influence of colour and the correlated colour temperature (CCT) of light on indoor thermal perception [[11], [12], [13], [14]] indicate that the colour of light can affect occupants’ thermal perception and, subsequently, energy consumption. Moreover, another crucial aspect of these glazings for interior design is the visual perception of indoor surface colours.

In order to analyse the non-visual effects and colour perception of light through glazing, it is necessary to consider the spectral power distribution (SPD) of transmitted light, which is not provided by traditional visualisation techniques. To address this, a Radiance-based tool, *Lark Spectral Lighting Simulation*, has been developed [15] to calculate the spectrum power distribution of light using nine channels by using the full spectrum of sky and material surfaces rather than the standard three RGB values, which is a significant improvement for investigating the impacts of daylight in the built environment.

The aim of the study is to evaluate the impact of daylight transmitted through one smart glazing (ChromoGenics) in comparison to a conventional clear glazing, employing the Lark Spectral Lighting Simulation tool. The study focuses on two aspects of transmitted daylight: (I) the non-visual effects of light (i.e., circadian, neurobehavioral, and neuroendocrine), and (II) the visual effects, focusing on the appearance of surface colours. Therefore, the main research questions are as follows:

• *How strong is the impact of smart glazing on the non-visual effects of transmitted daylight under different conditions throughout the year?*
• *How does the appearance of surface colours change with smart glazing?*

To the best of our knowledge, a study of electrochromic glazing considering all photoreceptors’ responses to light has not yet been performed using spectral lighting simulation techniques. This raises the third research question related to the methodology:

• *What are the benefits and limitations of using spectral lighting simulation techniques to study glazing behaviour in buildings?*

## 2 Theoretical background

### 2.1 Transmitted daylight through glazing

The spectral power distribution (SPD) of daylight in built environments not only causes colour variation due to time, location, and weather conditions but also varies based on the spectral properties of the glazing through which daylight enters buildings [16]. In several smart glazing studies, the subjective evaluation of daylight transmitted through tinted glazing has been examined. For instance, in an experiment conducted by Arbab et al. [17], participants viewed a space with different tinted glazing, demonstrating that the tint of the glazing significantly affects colour perception. Similarly, Arsenault et al. [18] investigated the effects of blue, neutral, and bronze glazing on daylight quality, arousal, and switch-on patterns for electric lights. Their evaluation of five light quality factors (visual comfort, naturality, pleasantness, precision of details and textures, and light level) using a scale model of an office room revealed a reduction in arousal with blue glazing, non-significant results in electric lights’ switch-on patterns, and a significant increase in the arousal levels of office workers with bronze window glazing.

Dubois et al. [19] investigated the visual perception of the interior and the view out in scaled rooms using six coated glazing materials under overcast skies for a north-oriented window. Despite variations in daylight transmittance (50%–79%), the results of light level measurements showed that glazing types with higher transmittance received more positive ratings for naturalness, beauty, pleasantness, and sharpness. In a study by Liang et al. [20], the human response to chromatic glazing was explored using bronze, blue, and clear thermochromic glazing. It was found that the bronze window glazing caused more errors in achromatic acuity tests than the blue or clear glazing. However, participants preferred the bronze glazing, as it provided a warm tint and a relatively natural rendering of the illuminated environment in which to reside and work.

The findings of a study by Angelo et al. [21] indicate that even transparent low-energy glazing, consisting of three layers of glass (two with low-emissivity coating), can cause colour distortions when compared to unfiltered daylight. This is due to the lower transmittance of the red and blue parts of the spectrum compared to the middle part (green-yellow), resulting in muted red and blue colours and a more monotonous appearance of the space when illuminated through such glazing as opposed to an open window. Similar questions prompted DeForest et al. [22] to explore the development of electrochromic glazing, which offers equal transparency across the whole range of visible light without compromising a building's daylighting level or aesthetic appeal.

A comprehensive study conducted by Salamati et al. [23] investigated the impact of thermochromic glazing on indoor visual comfort, colour quality, and non-visual effects of transmitted light, daylight availability, and artificial lighting load. The study concluded that lower glazing transparency softens daylight contrast and reduces the risk of glare in the workspace. The study found that thermochromic glazing was more effective in controlling glare at higher latitudes compared to clear and blue-tinted glazing, as the thermochromic glazing contributed to a 10% reduction in the daylight glare probability (DGP >0.4). Additionally, the non-visual effects of the glazing were assessed using ALFA (Adaptive Lighting for Alertness). The results revealed that thermochromic glazing exhibited the lowest Melanopic ratio (m/p) among the three glazing types, occurring with m/p < 0.9 in 32% of the cases. Clear glazing, despite having approximately 22% higher transmittance levels than blue-tinted glazing, showed a lower Melanopic ratio (clear glazing had a m/p < 0.9 in 18%, while blue-tinted glazing had a m/p < 0.9 in only 7% of the cases).

For a more comprehensive understanding of energy efficiency, thermal performance, and visual comfort concerning the integration of electrochromic windows, which are beyond the scope of the current study, please consult the following scholarly sources: [[24], [25], [26], [27], [28], [29], [30], [31], [32], [33], [34], [35]].

### 2.2 Non-visual effects of light

Light has the capacity to shift the phase of our circadian rhythm [36]. In the evening and at night, light may hinder falling asleep and interfere with the release of the night-time hormone melatonin [37]. Various factors, including timing, intensity, duration, wavelength, and prior light exposure, can impact circadian entrainment [[38], [39], [40]]. The non-visual effects of light (NIF) can be triggered by the light responses of photoreceptor cells in the retina, including the short-, medium-, and long-wavelength-sensitive cones, rod cells, and intrinsically photosensitive retinal ganglion cells (ipRGCs) [41]. Each photoreceptor type has distinct light-absorbing photopigments containing opsin proteins with sensitivity to specific portions of the light spectrum. Cones contain three opsins: S-opsin, M-opsin, and L-opsin, which enable colour vision by responding to blue, green, and red wavelengths. Rhodopsin, the primary opsin in rod cells, is essential for low-light vision [42]. Moreover, melanopsin, the photopigment found in ipRGCs, was discovered in the human eye by Provencio et al. [43]. Subsequent investigations to find the spectral sensitivity of human melatonin suppression to light [44,45] revealed its action spectrum peak sensitivity at 479 nm [46], in comparison to the visual system that is most sensitive to light at 555 nm, discovered decades earlier by Wright [47].

Researchers have devised a method for analysing and calculating the potency of light with respect to its non-visual effects on humans [48] based on α-opic action spectra for human responses to light (S-cone-opic (α: sc), M-cone-opic (α: mc), L-cone-opic (α: lc), Rhodopic (α: rh), and Melanopic (α: mel)). The approach defines Melanopic EDI (Equivalent Daylight Illuminance) as the illuminance produced by radiation of the standard illuminant D65 that provides an equal α-opic irradiance as test sources with respect to melanopsin activation [48]. Experts have established recommendations for Melanopic EDI levels to best support physiology, sleep, and wakefulness [49]. They recommend a minimum indoor light exposure of 250 lux of Melanopic EDI (measured vertically at the eye, approximately 1.2 m height) during the daytime. In the evening, light exposure should not exceed 10 lux, starting at least 3 h before bedtime, and the sleep environment at night should be as dark as possible, with a maximum Melanopic EDI of 1 lux.

Furthermore, St. Hilaire et al. [50] discuss how melatonin suppression and circadian phase resetting responses to light in humans are primarily controlled not only by melanopsin-containing ipRGCs but also by visual photoreceptors. During the initial stages of light exposure at low irradiance levels, circadian responses are primarily influenced by visual photoreceptors. However, for longer durations of light exposure at higher irradiance, melanopsin becomes the dominant circadian photopigment [50]. Their results indicate that the relative contribution of cone photoreceptors to melatonin suppression is strongest at the start of light exposure but decreases over the first 1–3 h, after which melanopsin becomes the primary contributor to melatonin suppression. Consequently, it is important for researchers to consider the responses of not only visual photoreceptors and Melanopic EDI but also the EDIs of other photoreceptors.

### 2.3 Spectral lighting simulation techniques

Inanici et al. [51] presented a multi-spectral simulation method for evaluating circadian lighting in built environments, considering local skies, the exterior environment, glazing optics, surface materials, interior design, and viewer location. The suggestion put forth was to enhance the accuracy of colour simulation in Radiance [52], a Monte Carlo ray tracing lighting simulation and visualisation system, by using the full spectrum of material reflectance instead of tristimulus values, as well as adopting channels instead of the traditional three RGB values, based on the method proposed by Ruppertsberg and Bloj [53,54]. The differences in simulating light and materials using RGB values or full spectrum data may impede the precise calculation of lighting colour-dependent lighting metrics and photoreceptors’ responses to light. The Lark simulation tool implements nine channels categorised as follows: B [b1 (380,422), b2 (422,460), b3 (460,498)], G [g1 (498,524), g2 (524,550), g3 (550,586)], R [r1 (586,650), r2 (650,714), r3 (714,780)] for wavelengths ranging from 380 to 780 nm. In comparison, ALFA [55], another spectral daylighting simulation tool, employs 81 channels. However, Lark, with its 9-channel approach, offers advantages over ALFA in this study due to its capacity to benefit from the Grasshopper/Rhino visual language programme environment, enabling flexibility and versatility in options. Notably, a study by Pierson et al. [56] demonstrated that Lark, although slower than ALFA, produces more accurate results under daylight conditions. The validation of Lark has also undergone examination, as documented in previous studies [[57], [58], [59], [60]].

The exploration of spectral lighting simulation for studying the non-visual effects and colour appearance of transmitted light through glazing is an area that requires further investigation. The utilisation of this technique holds potential for introducing new aspects to the field. Its potential to aid in better understanding the changes in daylight properties throughout the year can contribute to the creation of better and healthier built environments.

## 3 Methodology

Measurements in a controlled environment and simulations constitute the two components of this research method. To begin with, spectral irradiance measurements were conducted in the controlled environment to evaluate the transmitted daylight through the clear and electrochromic glazing in its maximum and minimum colorations under two distinct scenarios: clear and overcast sky conditions. On April 16, measurements were taken under a sunny sky. The measurements were conducted at 12:00 with 4 nm intervals within a wavelength range of 380 and 780 nm. Subsequently, on April 22, measurements were repeated under an overcast sky condition at the same time with the same wavelength range and intervals as mentioned earlier. Additionally, spectral radiance measurements for nine colour samples were performed under transmitted daylight through each glazing at the same time under the same sky condition. These measurements had 4 nm intervals (380–780 nm), with a 3-min gap between each colour sample measurement.

**Fig. 1:**
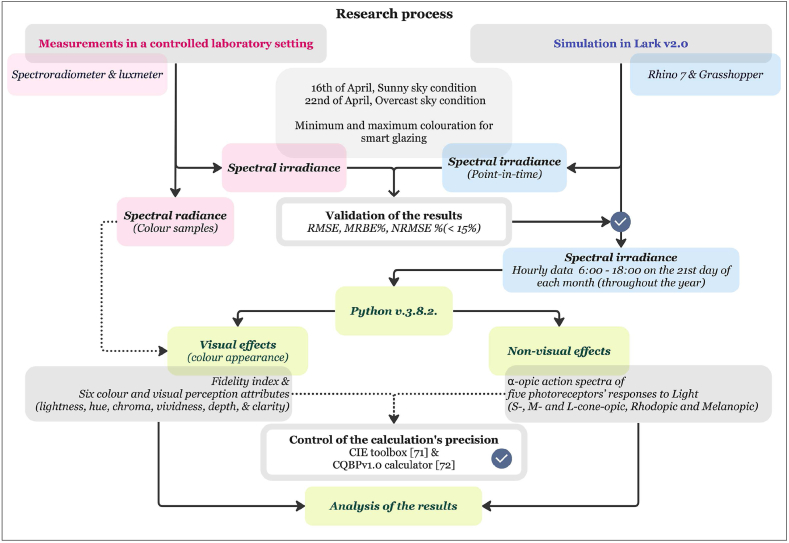
Overview of the research process.

In the second stage, point-in-time simulations of spectral irradiance were performed using the Lark spectral lighting simulation, specifically at 12:00 on April 16 and 22. The objective of these simulations was to evaluate the accuracy of the results by comparing them with the measured spectral irradiance data. This validation step ensures the reliability of the simulation process and its applicability throughout the year, accounting for various sky and weather conditions. Furthermore, hourly simulations were carried out on the 21st of each month, spanning a year, using the Lark point-in-time method. These simulations aimed to achieve a more comprehensive and representative understanding of the long-term effects of the behaviour of the glazing types under various sky conditions. Finally, the simulation results were analysed, and additional metrics were calculated using Python v.3.8.2. Fig. 1 provides an overview of the methodology.

### 3.1 Glazing type and colour samples

For this study, two types of glazing were considered: clear glazing (*Pilkington isulightherm, Optifloat clear* 4 mm*, Optitherm S1*) and smart glazing (*ChromoGenics Dynamic Glass 65 3G, clear*), while a window without any glazing was used as the control case. The estimated visual transmittance values of the glazings are presented in Table 1. Although illuminance measurements were collected under highly consistent lighting conditions, the assessment of visual transmittance using the ratio of the illuminance measured inside a space (illuminance in) to the external illuminance (illuminance ex) is not entirely precise due to variations in daylighting over time and the accuracy of the sensor positioning. Therefore, in the Results section, we provide more accurate measurements based on spectral data. Nonetheless, the estimated percentage of visual transmittance can still offer a general understanding of the glazing type.

**Table 1:** Estimated values of the Visual Transmittance of the glazing types.

Glazing	Visual Transmittance (T_vis_)	
	Sunny (maximum coloration)	Overcast (minimum coloration)
Clear Glazing	70%	72%
Smart Glazing	24%	65%

* Visual Transmittance (T_vis_): Illuminance (in)/Illuminance (ex).

To evaluate the influence of glazing on the visual appearance of colours, it was necessary to select a specific set of colour samples. The same colours that were previously identified by Arbab et al. [17], along with an additional colour sample that represents skin tone (sample 3), were chosen from the NCS standard samples. This selection resulted in a total of nine colour samples, which closely correspond to the eight standard CIE colours (TCS01-08) outlined in Table 2 and one additional colour (TCS13).

**Table 2:**
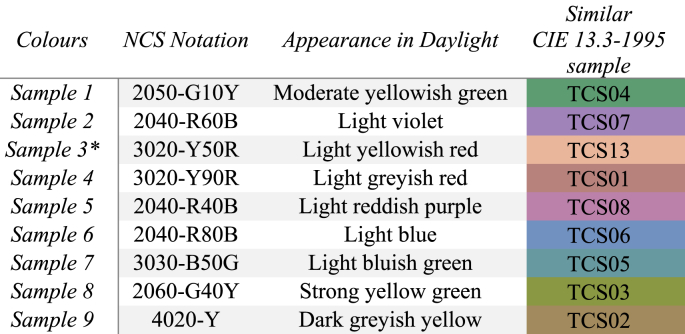
Colour Samples.

### 3.2 Experimental setup

**Fig. 2:**
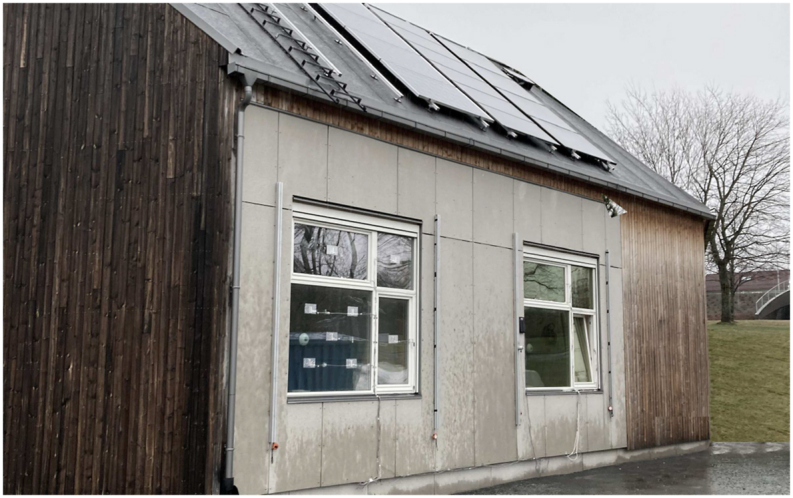
The ZEB Test Cell Laboratory, with the test room window visible on the right side of the image (63° N, 10° E).

The experiment was conducted in a controlled environment at the ZEB Test Cell Laboratory (Fig. 2, coordinates: 63° N, 10° E) located at the Norwegian University of Science and Technology (NTNU) in Trondheim, Norway. The test room featured a large window facing south, equipped with ChromoGenics glazing connected to a voltage source and allowing for adjustable coloration, ranging from minimum (bleach state) to maximum. The window had a portable section that was kept fully open during the experiment. Measurements were carried out using three identical boxes constructed with white foamboard as scale models of a larger room. These boxes measured 40 cm by 40 cm with a depth of 70 cm. They were positioned adjacent to the window frame inside the test room. The first box was situated in front of the largest part of the window (referred to as SG: “smart glazing”). The second box was positioned in front of the Plingthon clear glazing that was temporarily placed in the open part of the window (referred to as CG: “clear glazing”). The third box was placed in front of the open section, which was not equipped with any glazing (referred to as NG: “no glazing”) and served as the control case.

### 3.3 Measurements

The study employed a systematic method to gather comprehensive spectral data on irradiance, radiance, and material reflectance. To ensure accuracy, measurements were conducted using the SpectraScan® Spectroradiometer PR-655 under stable lighting conditions. The spectroradiometer was instructed to take three consecutive measurements and report the average results, especially in measurements where low light levels are being tested. Additionally, the illuminance was measured using the Hagner luxmeter Model EC1. Professional NCS colour samples were employed to ensure uniformity and the absence of unwanted particles or contaminants. These samples, representing nine selected colours, were placed horizontally, one at a time, in the middle of boxes during measurements.

The spectral radiance measurements were taken with the spectroradiometer positioned at a 50-cm distance and a 45-degree angle away from the perpendicular direction of the sample's surface. The spectroradiometer was directed at the samples to capture radiance data across the visible spectrum (380–780 nm) with 4 nm intervals, while the measurements with the luxmeter ensured the same level of light for all the samples. Exposure times were adjusted using the Adaptive Sensitivity® algorithm to ensure accurate measurements, with an average of 220 msec for sunny skies and 1236 msec for overcast skies. Additionally, irradiance measurements were conducted in the middle of all three boxes, 40 cm away from the glazing surface, capturing data over the wavelength range of 370–780 nm with 4 nm intervals. The spectral reflectance of the material surfaces was determined by comparing the measured spectral radiance with a known reference (D65), using the Reflectance Standard (RS-3), with an absolute reflectance of 99% (±1%), as the source, allowing for the calculation of the relative and absolute reflectance of materials at each wavelength. The measurement instrument carried out the automated calculation process.

To ensure comprehensive data, measurements were repeated in both sunny and overcast conditions, accounting for the colour variation in the tinted ChromoGenics glazing. For the sunny sky, the glazing was adjusted to its maximum coloration, while for the overcast sky, it was set to its minimum coloration (bleach state) (Table 3).

**Table 3:** Two sky conditions (sunny and overcast, with maximum and minimum coloration of smart glazing).

Focus	Sunny (maximum coloration)	Overcast (minimum coloration)
Non-visual effects		
Indoor colour perception		

### 3.4 Simulation

For this study, Lark [15] in the Grasshopper environment in Rhino 7 software was chosen as one of the currently available spectral lighting simulation tools. Instead of directly predicting the sky spectrum, Lark models the sky spectrum using user-selected Spectral Power Distributions (SPDs) [58]. This paper employs the latest version of Lark v2.0, which includes additional features developed by Pierson and Gkaintatzi-Masouti [61,62].

To perform the simulation of spectral irradiance over a year-long period, the point-in-time simulation template was modified using Grasshopper code to automatically compute hourly data. Given that the longest and shortest days over a year correspond to the longest and shortest daylight hours, our focus was on collecting data for the summer solstice (June 21) and winter solstice (December 21). Therefore, the hourly simulations were set for the 21st of each month (January to December) from 6:00 to 18:00. Consequently, the simulation data for June represents the summer solstice, December represents the winter solstice, and March and September represent equinoxes. The calculated α-opic EDIs (Equivalent Daylight Illuminances) derived from the spectral irradiance simulation data on 12 selected dates (one for each month) serve as a useful example to understand the influence of smart glazing on the non-visual effects of transmitted daylight under various sky conditions.

**Fig. 3:**
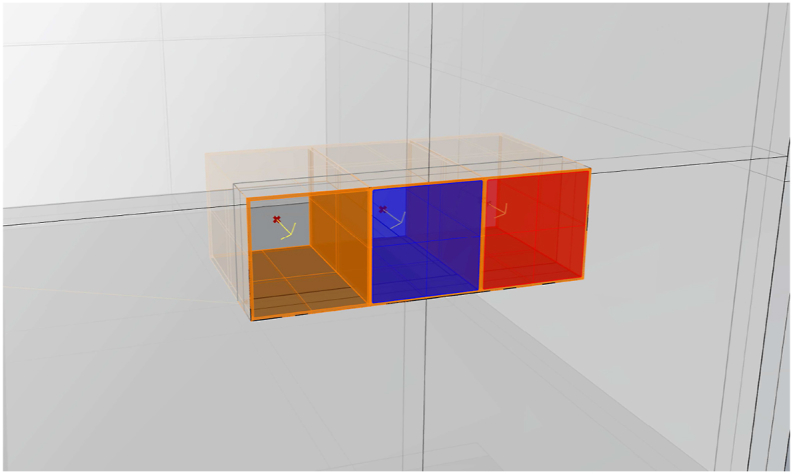
The simulation model showing the boxes for No glazing, Clear and Smart glazing, with the position of sensors in the middle of each box and 40 cm away and directed towards the glazings.
• The spectroradiometer was used to measure the spectral reflectance (see 3.3 Measurement), which represents one attribute of the optical properties of material surfaces. Measurements were conducted in a wavelength range of 380–780 nm with 4 nm intervals, and the obtained values were imported into Lark.
• The spectral transmittance of clear glazing was obtained from the IGDB, the International Glazing Database [63], whereas the spectral transmittance of smart glazing was estimated using the measurements of the spectral irradiance transmitted in maximum and minimum coloration states in comparison to direct daylight without glazing. The software Optic0.6 [64] generated a *.usr* file with the calculated optical properties of the clear glazing and two *.usr* files containing the estimated optical properties of ChromoGenics glazing, in maximum and minimum colorations. These resulting *.usr* files were then imported into Lark.
• To ascertain the most appropriate weather file (*.epw*) between the two available options, which cover the periods 2004–2018 and 2007–2021, a brief evaluation was conducted. The analysis aimed to assess the weather and sky conditions for Trondheim (63° N, 10° E) at the measurement time of 12:00 on April 16 and 22. It was found that the weather file for the period of 2004–2018, obtained from the Lawrie and Crawley [65] database, closely matched the observed sky conditions, with a cloud cover of 0 on April 16 and an overcast sky condition with a cloud cover of 9 on April 22 (on a scale of 0–10; 10 total coverage). Consequently, this specific weather file was selected as the input for the simulation process.
• The position and direction of each sensor for measurements inside each box were defined. Specifically, one sensor for each glazing is in the middle of the box, 40 cm away from the glazing (Fig. 3).
• The model was calibrated and validated using two sets of measured data: one for a sunny sky condition and the other for an overcast sky condition.
• The default spectral power distribution (SPD) of the sky was utilised, with only the diffuse part set to the CIE standard illuminant D65 [66].
• The Radiance simulation parameters are presented in Table 4.

To ensure the precision of the simulation model, the following measures were implemented:

• The 3D model of the test room was developed in Rhino 7 (educational licence) based on the in-situ measured values (Fig. 2, Fig. 3).

**Table 4:** Radiance simulation parameters.

-ab ambient bounces	-ad ambient divisions	-as ambient super-samples	-ar ambient resolution	-aa ambient accuracy	-Iw limit weight of each ray
3	1024	500	100	0.1	0.0001

## 4 Analysis method

The spectral irradiance output generated by Lark was used in Python to calculate several metrics, including illuminance (lux), correlated colour temperature (CCT), chromaticity coordinates (CIEx and CIEy) [[67], [68], [69]], values for α-opic equivalent daylight (D65) illuminance (EDI), efficacy of luminous radiation (ELR), daylight efficacy ratio (DER) [48], and fidelity index (R_F_) [70]. To ensure the reliability of the Python code in computing α-opic EDIs values, the same random spectral irradiance data was cross-verified using the CIE toolbox [71] and the CQBPv1.0 calculator [72].

### 4.1 Non-visual effects

The analysis for non-visual effects of light in this study accounts for α-opic values using the CIE-S026 method [48], adopting the α-opic action spectrum (the relative spectral sensitivity of one of the five human α-opic photoreceptors’ responses to light) (Table 5).

**Table 5:** The non-visual metrics and calculation methods [48].

Metric and definition	Formula
**α-opic irradiance** (μW.cm-2)	α-opic irradiance = ∫ spectral irradiance * α-opic action spectrum * dλ
**α-opic ELR** α-opic efficacy of luminous radiation, (mW.lm-1)	α-opic ELR = α-opic irradiance/illuminance
**α-opic DER** α-opic daylight (D65) efficacy ratio	α-opic DER = α-opic ELR/α-opic ELR for daylight (D65)
**α-opic EDI** α-opic equivalent daylight (D65) illuminance, (lux)	α-opic EDI = α-opic irradiance/α-opic ELR for daylight (D65)

### 4.2 Visual aspects

The visual aspect examines the colour appearance of the test samples based on the calculation method recommended by the CIE colorimetry parts 1, 3, and 4 [[67], [68], [69]]. To analyse the differences in colours, six attributes have been considered: Lightness, Hue, Chroma, Vividness, Depth, and Clarity [69,73] (Table 6).

**Table 6:** Definitions of the attributes of visual perception and colour and calculation formula.

Attribute and its definition [73]	Formula
**Lightness** (an attribute of visual perception) Compares a colour to a neutral tone ranging from black to white.	[69]: L*=116f(YYn)−16
**Hue** (an attribute of visual perception) The appearance of an area as red, yellow, green, or blue, or as a combination of adjacent pairs of these colours in a closed ring.	[69]:hab=arctan(b*a*)
**Chroma** (an attribute of colour) The distance from a neutral colour of the same lightness.	[69]: $C_{ab}^{*}=\sqrt{{\left(a^{*}\right)}^{2}+{\left(b^{*}\right)}^{2}}$
**Vividness** (an attribute of colour) The departure of a colour from neutral black. Colours that are “Cleaner” or “brighter” increase the CIELAB vividness.	[73]: $V_{ab}^{*}=\sqrt{{\left(L^{*}\right)}^{2}{+{\left(a^{*}\right)}^{2}+\left(b^{*}\right)}^{2}}=\sqrt{{\left(L^{*}\right)}^{2}+{\left(C_{ab}^{*}\right)}^{2}}$
**Depth** (an attribute of colour) The departure of a colour from neutral white. Colours that are “Darker” or “stronger” increase the CIELAB depth.	[73]: $D_{ab}^{*}=\sqrt{{\left(100-L^{*}\right)}^{2}+\left(a^{*}\right)+{\left(b^{*}\right)}^{2}}$
**Clarity** (an attribute of colour) The difference between a colour and its background colour.	[73]: $T_{ab}^{*}=\sqrt{{\left(L_{bk}^{*}-L^{*}\right)}^{2}+{\left(a_{bk}^{*}-a^{*}\right)}^{2}+{\left(b_{bk}^{*}-b^{*}\right)}^{2}}$ (bk: background)

L* (CIELAB lightness), a*, b* (CIELAB a*, b* coordinates); for the definition of Y and Y_n_, see Ref. [69].

## 5 Results

### 5.1 Spectral irradiance and spectral radiance

**Fig. 4:**
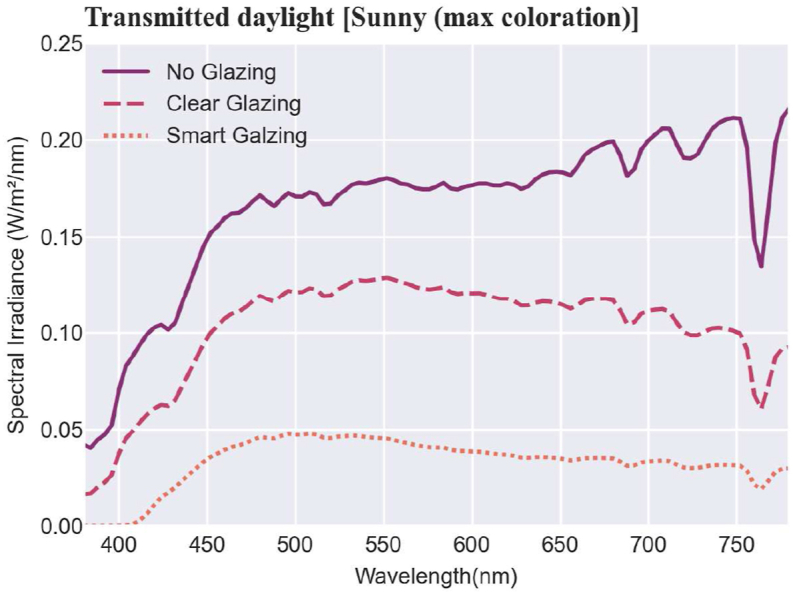
Measured absolute values of the spectral irradiance of the transmitted daylight (note: the scale of the ‘y’ axis is 0–0.25 W/m2/nm) in the sunny sky condition.

**Fig. 5:**
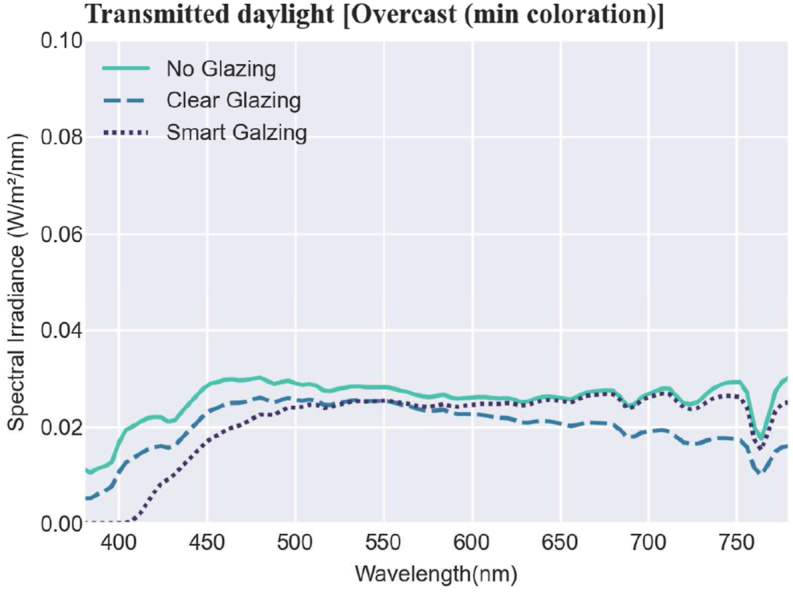
Measured absolute values of the spectral irradiance of the transmitted daylight sky (note: the scale of the ‘y’ axis is 0–0.1 W/m2/nm) in the overcast sky condition.

Spectral power distribution (SPD) values are the fundamental data used in this paper to calculate the visual and non-visual effects of daylight transmitted through glazing. The measured spectral irradiance absolute values of transmitted daylight are shown in Fig. 4 in a sunny sky condition and Fig. 5 in an overcast sky condition (the upper curves: no glazing, the middle: clear glazing, and the bottom: smart glazing in maximum and minimum colorations for sunny and overcast skies, respectively).

Fig. 4, Fig. 5 show that the spectral irradiance of the transmitted daylight is varied all over the spectrum in different scenarios. During the overcast condition, the smart glazing was in a bleach state with 65% visual transmission, which is almost similar to the clear glazing with 72% (Fig. 5). In comparison, under the sunny condition, due to the low level of visual transmittance (24% for the smart glazing in the maximum coloration), the spectral irradiance values are drastically smaller, and the smart glazing filters any light with a shorter wavelength than 400 nm in both the maximum and minimum colorations. Note that for visibility, in Fig. 4, Fig. 5, the values are shown in different scales for the y axis.

**Fig. 6:**
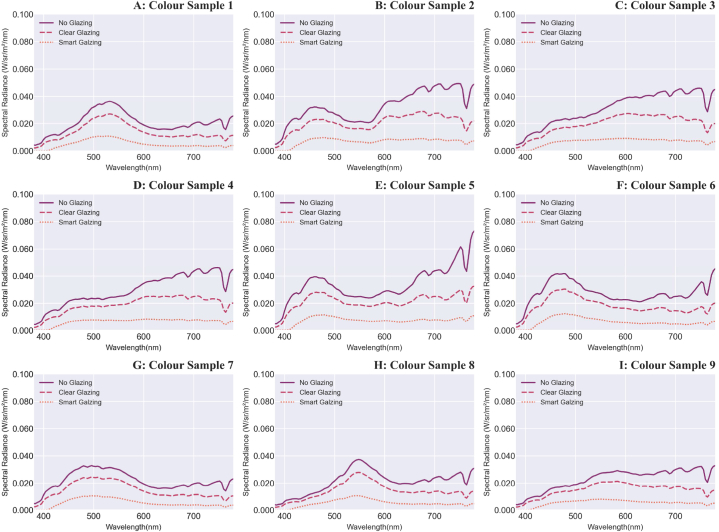
A–I: Measured absolute values of the spectral radiance of the nine colour samples (note: the scale of the ‘y’ axis is 0–0.1 W/sr/m^2^/nm) under daylight transmitted through glazing types in the sunny sky.

**Fig. 7:**
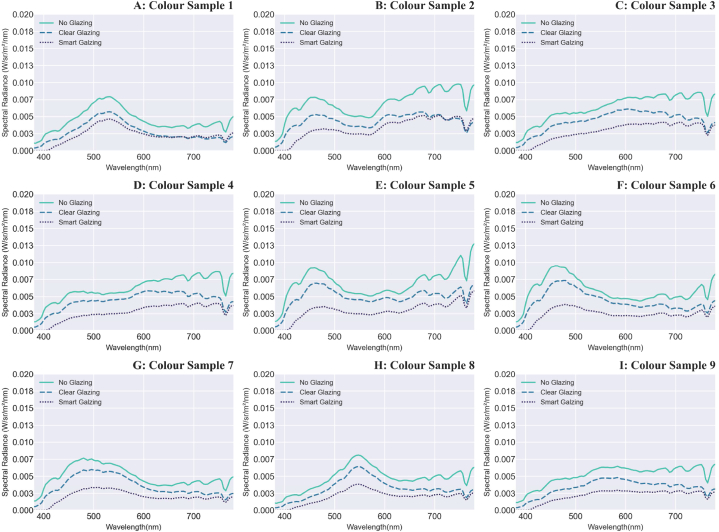
A–I: Measured absolute values of the spectral radiance of the nine colour samples (note: the scale of the ‘y’ axis is 0–0.02 W/sr/m^2^/nm) under daylight transmitted through glazing types in the overcast sky.

The absolute values of spectral radiance of nine colour samples under sunny and overcast sky conditions are illustrated in Fig. 6 (A-I) and Fig. 7 (A-I). The values of each sample vary for each glazing type at different magnitudes, and to understand these variations, further colour attributes are discussed in the following sections.

### 5.2 Non-visual effects (NIF: non-image-forming responses to light)

#### 5.2.1 Measurement

**Fig. 8:**
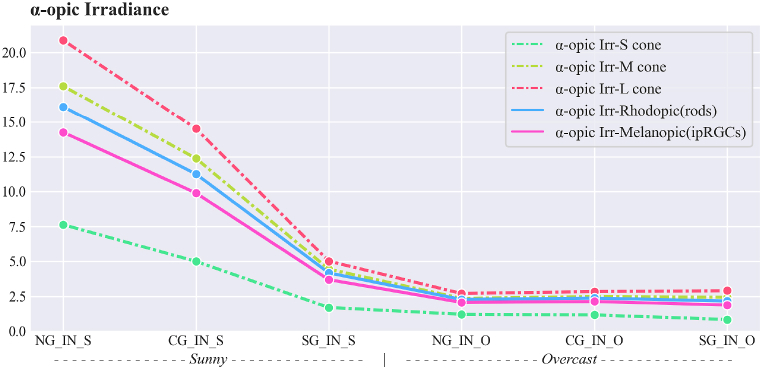
α-opic irradiance (μW.cm^−2^).

**Fig. 9:**
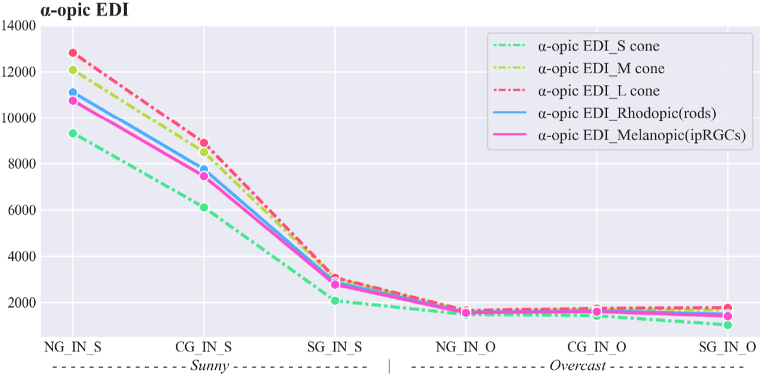
α-opic EDI (Equivalent Daylight (D65) Illuminance, (lux)).

The non-visual effects of daylight were analysed using the spectral irradiance of daylight transmitted through the glazing (see Table 5). The α-opic values for the responses to light of the five photoreceptors are presented in Fig. 8, Fig. 9 for both sunny and overcast sky conditions, with maximum and minimum coloration for smart glazing.

As expected, NG in the sunny sky condition has the highest values of α-opic irradiance and EDI, while SG in the overcast sky condition has the lowest values.

**Fig. 10:**
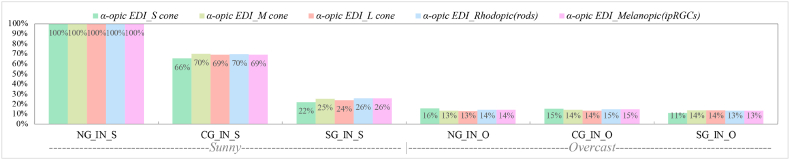
α-opic EDI efficacy rate in comparison to the window without glazing (NG) in the sunny sky.

Fig. 10 presents the α-opic EDI efficacy rate for S-*M*-L cones, Rhodopic and Melanopic, respectively. Intriguingly, the values of α-opic EDI for smart glazing in maximum coloration in the sunny sky condition decrease to nearly the level observed in the overcast sky condition. For example, α-opic EDI Melanopic in Smart glazing (maximum coloration) is 26% of α-opic EDI Melanopic for the window with no glazing in the sunny sky. Moreover, among different photoreceptors, s-cone has the highest decrease in values.

CCT values for transmitted daylight in the sunny sky condition are NG: 4853K, CG: 4952K, SG_max_: 5438K, and in the overcast sky condition, NG: 5703K, CG: 5625K, and SG_min_: 4574K.

#### 5.2.2 Simulation

##### 5.2.2.1 Validation

**Fig. 11:**
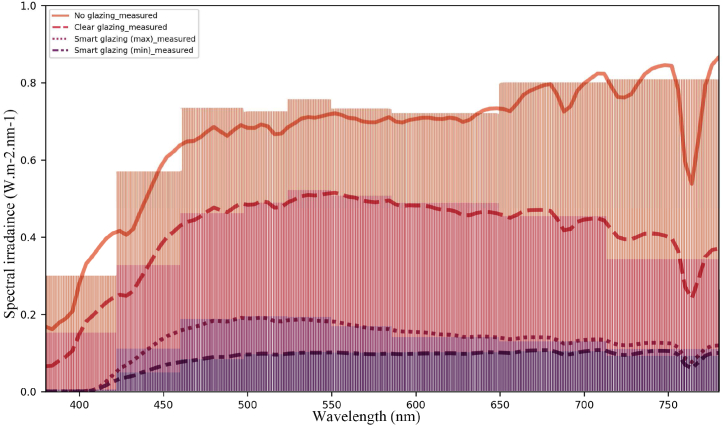
The spectral irradiance (absolute values) data from measurements (line-plot) and simulation (bar-plot).

To evaluate the accuracy of the acquired data, the spectral irradiance was validated using actual radiometric measurements taken on April 16 (sunny sky) and April 22 (overcast sky) at 12:00 (Fig. 11). To assess the similarities or distinctions between data series, statistical error metrics including RMSE (relative root mean square error), MRBE (median relative bias error), and NRMSE (normalised root mean square error) were calculated with the absolute value of spectral irradiance across the visible range (380–780 nm), adopting the equations utilised by Pierson et al. [60].

The RMSE (equation (1)) measures the variation between the simulated and measured spectra, indicating the extent of fluctuation [74]. However, the RMSE solely quantifies the overall error magnitude and not bias, as it doesn't discern between positive and negative errors [75]. Addressing this, MRBE (equation (2)) evaluates the central tendency of relative bias errors between predictions and actual values, signifying whether the simulated spectrum surpasses or falls short of the measured one. Additionally, NRMSE normalises RMSE relative to mean actual values as a percentage, facilitating standardised performance comparison by accounting for both scale and variability.

(1) $$RMSE\left[-\right]=\sqrt{\frac{\sum_{i=1}^{n}{\left(I_{simulated{,}_{i}}-I_{measured{,}_{i}}\right)}^{2}}{n}}$$

(2) $$MRBE\left[\%\right]=median\left[\frac{I_{simulated{,}_{i}}-I_{measured{,}_{i}}}{I_{measured{,}_{i}}}\times 100\right]$$

(3) $$NRMSE\left[\%\right]=\frac{RMSE}{{mean[I}_{measured{,}_{i}}]}\times 100$$

In equations (1), (2), (3) $I_{simulated{,}_{i}}$ is the simulated spectral irradiance at the wavelength *i*; $I_{measured{,}_{i}}$ is the measured spectral irradiance at the wavelength *i;* and the ${mean[I}_{measured,}]$ represents the average spectral irradiance across all the measured wavelengths.

The data from Table 7 reveals that the simulation slightly underestimated the spectral irradiance for Smart glazing with the maximum coloration, as evident from the negative MRBE value. This same scenario also exhibits the highest NRMSE variation. Regarding acceptable NRMSE, ASHRAE Guideline 14 [76] recommends maintaining NRMSE (referred to as CV(RMSE): Coefficient of Variation of Root Mean Square Error) below 15% for monthly calibration and 30% for hourly calibration data in computer models. For spectral irradiance, Pierson et al. [60] count the NRMSE value below 20% as an acceptable range. In line with these benchmarks, the values presented in Table 7 underscore that the simulated model is in good agreement with the measured data (NRMSE <15%).

**Table 7:** RMSE, MRBE%, and NRMSE% for absolute values.

Indices	RMSE	MRBE %	NRMSE
NG	0.0172	4.19%	10.44%
CG	0.0098	1.51%	9.65%
SG_max_	0.0036	−0.61%	10.92%
SG_min_	0.0020	1.25%	9.53%

##### 5.2.2.2 The non-visual effects over the course of a year

Vertical illuminance and CCT (K) (McCamy's cubic approximation) are presented in Table 8 for windows with no glazing (NG), clear glazing (CG), smart glazing in maximum coloration (SG_max_), and smart glazing in minimum coloration (SG_min_).

**Table 8:**
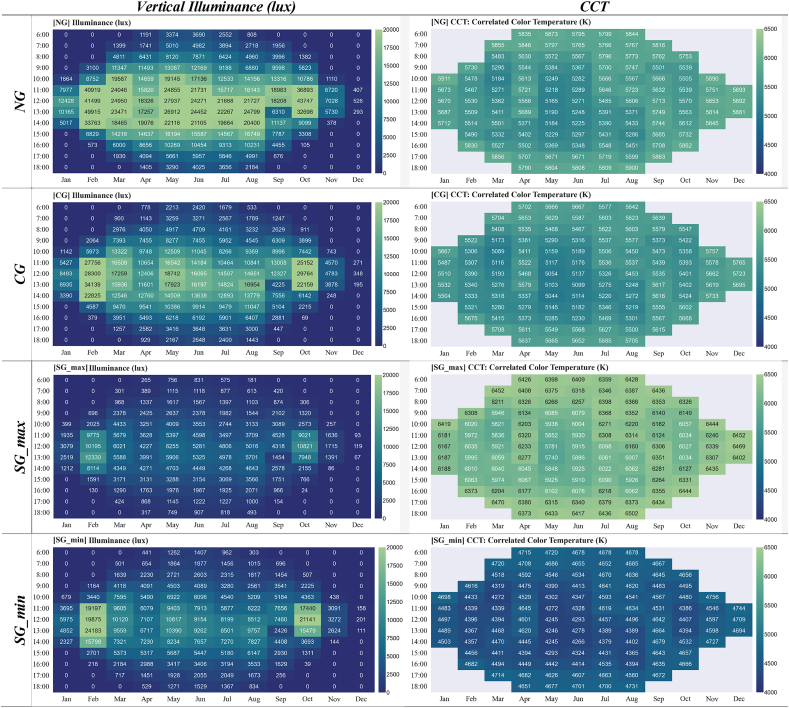
Vertical Illuminance (lux) and CCT (K) at the centre of each box facing directly towards glazing (see Fig. 3).

**Fig. 12:**
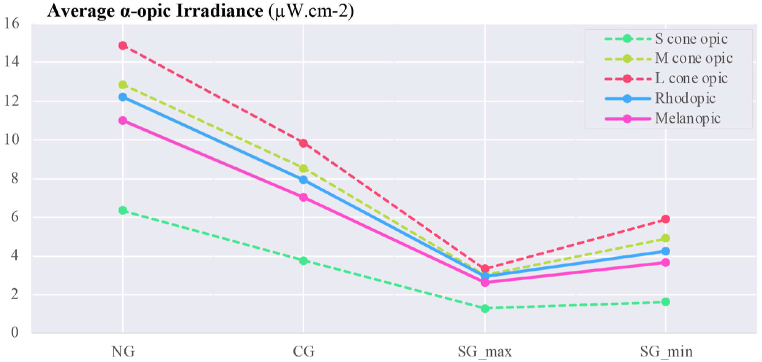
Average α-opic irradiance (μW/cm^2^) over a year for simulated data.

**Fig. 13:**
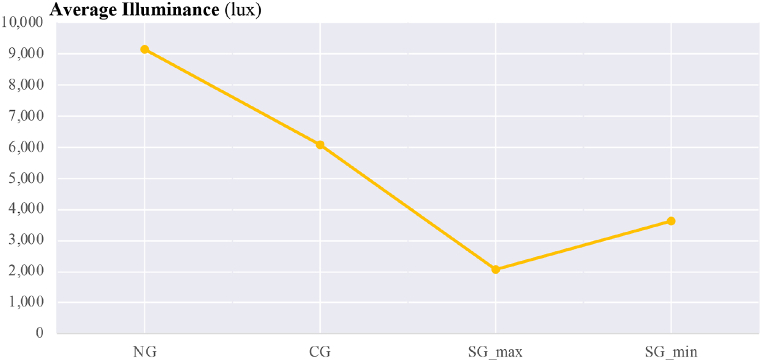
Average vertical illuminance (lux) over a year for simulated data.

**Fig. 14:**
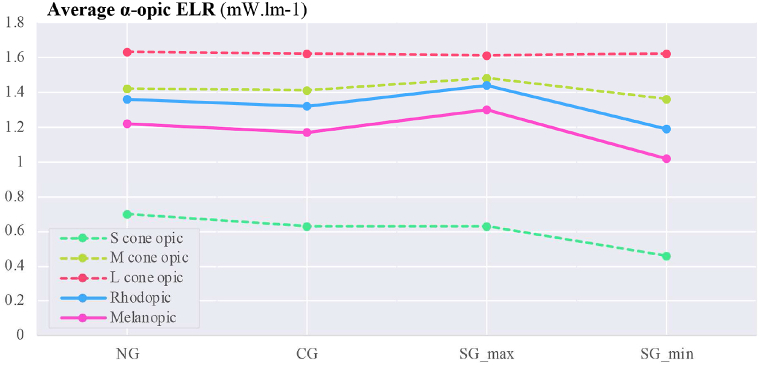
Average α-opic ELR (α-opic efficacy of luminous radiation_ mW/lm) over a year for simulated data.

**Fig. 15:**
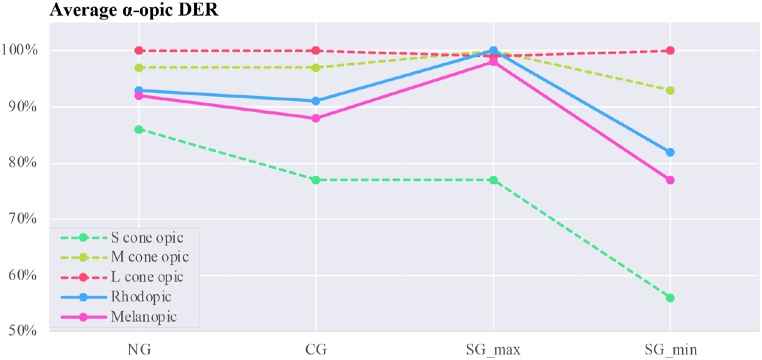
Average α-opic DER (α-opic daylight (D65) efficacy ratio_%) during a year for simulated data.

The overall assessment of calculated parameters for average vertical illuminance, α-opic irradiance, α-opic ELR, and α-opic DER for simulated data (Fig. 12, Fig. 13, Fig. 14, Fig. 15) revealed the lowest α-opic efficacy of luminous radiation (ELR) and α-opic daylight (D65) efficacy ratio (DER) for S-cones when daylight is transmitted through the studied glazing types. The α-opic ELR/DER varied least for L-cones for glazing type and sky conditions and most for the α-opic ELR/DER of S-cones (NG (overcast) 0.73/90% to SG_min_ (overcast) 0.46/56%) and Melanopic (SG_max_ (sunny) 1.3/98% to SG_min_ (overcast) 1.02/77%).

The diagram for α-opic EDIs over the course of a year is presented in Table 9. The Melanopic EDIs at noon (12:00) on the summer solstice are 21,555 lux (NG); 13,644 lux (CG); 5,006 lux (SG_max_); and 6,776 lux (SG_min_). In comparison, at the same time on the winter solstice, these values are 499 lux, 317 lux, 120 lux, and 160 lux for NG, CG, SG_max_, and SG_min_, respectively. Considering the Melanopic EDIs at noon (12:00), although the lowest values occur during the winter solstice, the highest values are not at the summer solstice but in February and October for NG: 37,897 and 40,117 lux, for CG: 24,885 and 26,181 lux, for SG_max_: 9,774 and 10,372 lux, and for SG_min_: 15,071 and 16,046 lux correspondingly.

**Table 9:**
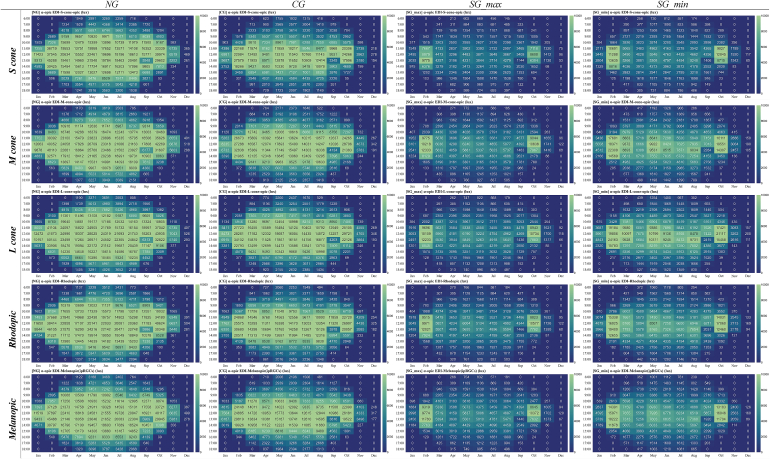
The α-opic EDI over the course of a year on the 21st of each month from 6:00 to 18:00 (simulation data).

### 5.3 Visual effects (IF: image-forming responses to light)

To evaluate the changes in appearance of surface colours under daylight transmitted through the studied glazing types, we employed the CIE 1976 L*a*b* [69] colour space, in which the distance between points represents the difference in colour appearance. The X, Y, and Z tristimulus values of colour samples were calculated using the colour-matching functions of the CIE 1964 standard colorimetric system (2° standard observer) using measured spectral radiance values [68].

**Fig. 16:**
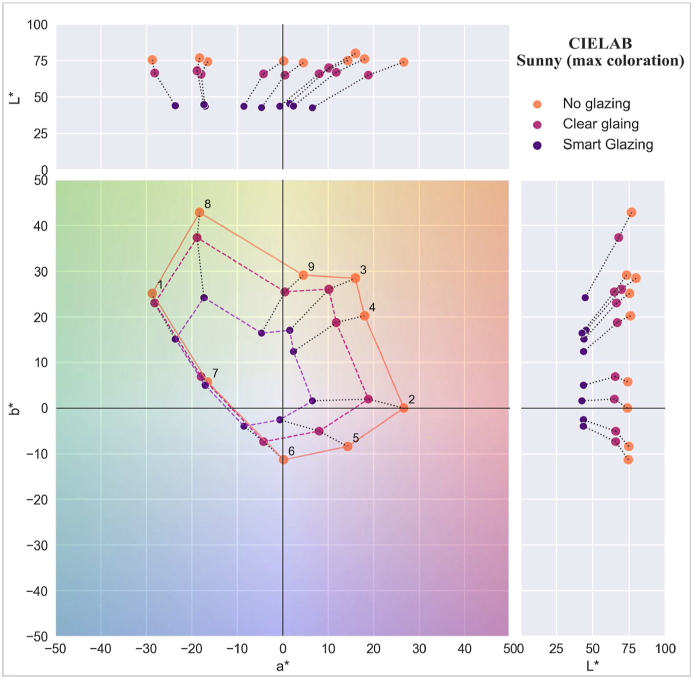
CIELAB a* and b* values for a sunny sky.

**Fig. 17:**
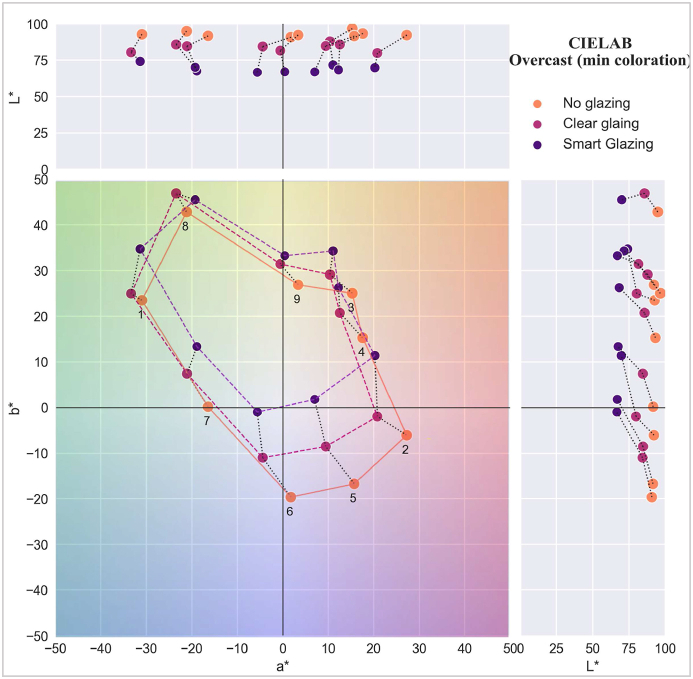
CIELAB a* and b* values for an overcast sky.

The CIELAB for sunny and overcast skies is presented in Fig. 16, Fig. 17. The numbers in the diagrams correspond to the colour samples (refer to Table 2), while the polygons represent the colour variation under daylight transmitted through the three scenarios. The diagrams show the a* and b* values of each colour sample and the overall direction of colour shift, while the top and right diagrams present lightness (L*-a* and L*-b*).

In Fig. 16 (sunny sky condition), the polygon depicting colours in the scenario without glazing exhibits the largest area, followed by Clear glazing and Smart glazing, which signifies the extent of variations observed across distinct scenarios. For instance, the blue-green colour sample (number 7) remains similar in all three scenarios, while the colour samples with blue-green, green, and yellow (numbers 1, 8, 9, 3, and 4) observe a lesser degree of colour variation with Clear glazing compared to Smart glazing. Even for clear glazing, there is considerable colour shift in red and purple colours (numbers 2, 5, and 6). The predominant shift direction for several colours (numbers 3, 4, 2, 5, and 6) is towards green.

In Fig. 17 (overcast sky condition), the overall colour shift leans towards yellow, diverging from the green-blue shift observed in the sunny sky. Additionally, the area of the connecting polygon exhibits milder changes than the sunny condition, with less pronounced colour shifts observed, particularly in the yellow and orange areas. For instance, the light green, yellow, and orange colour samples (numbers 8, 9, and 3) have similar appearances in daylight transmitted through Smart and Clear glazing, while, similar to sunny conditions, the maximum colour shift is for purple samples (numbers 2, 5, and 6) for both smart and clear glazing. Whereas blue and green samples (numbers 7 and 1) have a colour shift towards green and yellow, the appearance of these colours stays similar for clear glazing and no glazing.

**Fig. 18:**
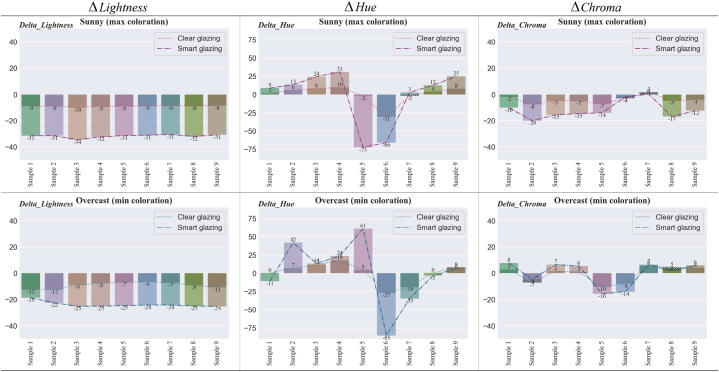
Difference in Lightness, Hue, and Chroma for the colour samples in Clear and Smart Glazing from No Glazing.

**Fig. 19:**
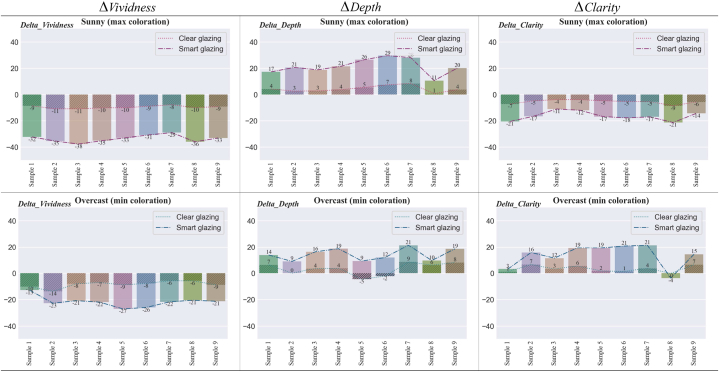
Difference in Vividness, Depth, and Clarity for the colour samples in Clear and Smart Glazing from No Glazing.

To explore the changes in the appearance of colour samples associated with each type of glazing, we analyse six attributes: lightness, hue, chroma, vividness, depth, and clarity (refer to Table 6). The magnitude of changes in each attribute is presented in Fig. 18, Fig. 19. The change refers to the difference between the control case (no glazing) and the Clear or Smart glazing. For example, the ΔHue values in Fig. 18 for Smart glazing show the difference between hue angles for Smart glazing and hue angles without any glazing (control case).

The diagram of ΔLightness shows that the change of lightness is almost uniform for all colour samples, with an average of −9 for Clear glazing and an average of −31.5 for Smart glazing. This means that under sunny skies, the colour samples are 3.5 times lighter in clear glazing compared to smart glazing (maximum coloration). Under overcast skies, the colour samples are 2.3 times lighter in Clear glazing than Smart glazing with minimum coloration (an average of −9 for Clear and −21 for Smart glazing).

Next, ΔHue's diagrams illustrate the difference in hue angles between Clear or Smart glazing and no glazing. The largest hue changes are for colour samples 5 and 6 (blue and purple) in both sunny and overcast sky conditions. The next largest shift in hue is in samples 3 and 4 (skin tone and brown) in the sunny sky and samples 2 (purple) and 7 (blue green) in the overcast sky. The changes in hue angle for colour sample 5 in the sunny and overcast skies are in opposing directions (towards red in the sunny and towards blue during an overcast sky).

Last, ΔChroma during a sunny sky (colour samples 2 and 8, purple, and light green) and during the overcast sky (colour samples 5 and 6, purple, and blue) have the largest shifts in chroma compared to no glazing. For all three attributes, lightness, hue, and chroma, the shifts are larger for Smart glazing than for Clear glazing.

Fig. 19 shows the attributes that consider the ambient colours: vividness (from white), depth (from black), and clarity (from the background colour). The average shift in vividness for Clear glazing is −10, while in overcast this value is −7.6, and for Smart glazing it is −29.6 for the sunny sky and −21.7 in the overcast sky. The depth of the colours increases for both glazing types under both sunny and overcast skies. However, there are two exceptions where their depth decreases for samples 5 and 6 under the overcast sky. Finally, the clarity of colour samples is reduced under the sunny sky when compared to the window without any glazing. However, in the overcast sky condition, the clarity of the colour samples is enhanced in relation to their background colour.

To conclude, the fidelity index (R_f_) [70] derived from the measured spectral irradiance of transmitted daylight (presented in Table 10) offers an overview of the impact of glazing type on the appearance of surface colours. The fidelity index [70] indicates that the lowest value is 92 for Smart glazing in maximum coloration, while in minimum coloration, the index increases to 96.

**Table 10:** Fidelity index for measured spectral irradiance.

Sky condition	Sunny			Overcast		
Glazing types	NG	CG	SG_max	NG	CG	SG_min
Fidelity index (R_f_)	98	95	92	98	97	96

Furthermore, the average fidelity indices obtained from the simulation process using spectral irradiance results showcase the long-term impact of glazing types. Over the course of a year, the average fidelity indices are 98 (NG), 97 (CG), 90 (SG_max)_, and 94 (SG_min_). These values further emphasise the influence of glazing type on maintaining the fidelity of surface colours.

## 6 Discussion

### 6.1 Non-visual effects

The results indicate that different types of studied glazing had distinct non-visual effects on the responses of the five photoreceptors to transmitted daylight throughout the year, as indicated in Table 9. The α-opic EDIs (Fig. 9) for measured values also revealed variance between the daylight transmitted through Clear and Smart glazing with ChromoGenics glazing (maximum and minimum coloration) under sunny and overcast skies. This section specifically addresses two main areas: identifying the potential causes of the observed variations and assessing the adequacy of light intensity and duration according to the recommendations.

**Fig. 20:**
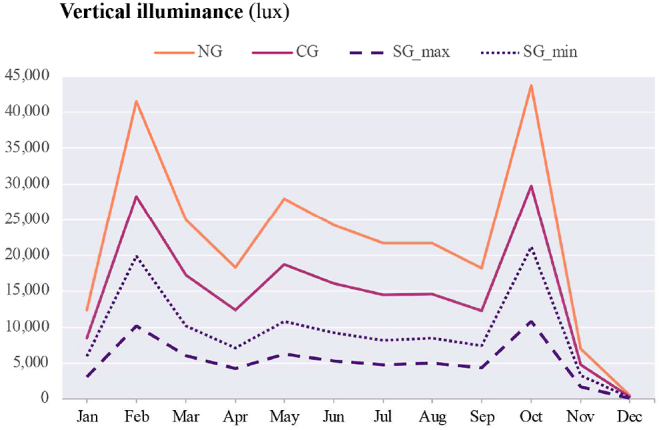
Vertical illuminance variation during a year at 12:00 (local noon).

**Fig. 21:**
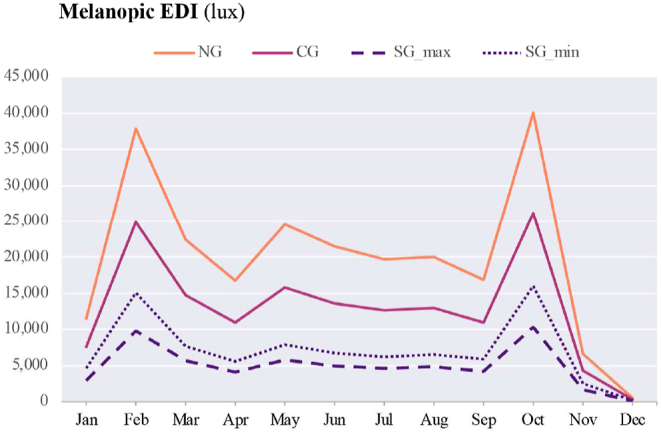
Melanopic EDI variation during a year at 12:00 (local noon).

Firstly, considering the sun's altitude is quite helpful for comprehending the potential cause of variation in maximum and minimum vertical illuminance values. Fig. 20, Fig. 21 demonstrate how changes in vertical illuminance and Melanopic EDI differ by month of the year at local noon (12:00). In December, the lowest vertical illuminance values are observed for the window without any glazing (526 lux), Clear glazing (348 lux), and Smart glazing in maximum and minimum coloration (119–201 lux), corresponding to a sun altitude of 3.70° at the experiment's location (63° N, 10° E). The minimum Melanopic EDI values at this time are 499 lux for NG and 317 lux for CG and range from 117 lux (max tint) to 120 lux (min tint) for SG. On the other hand, the highest vertical illuminance values at local noon occur in February and October, corresponding to sun altitudes of 15.90° and 16.48°. For example, during October, the vertical illuminance at local noon for NG and CG is 43,747 and 29,764 lux, and for SG (with minimum to maximum tint), it ranges from 21,141 to 10,821 lux. While the Melanopic EDI values are 40,117 and 26,181 lux and range from 16,046 lux (min tint) to 10,372 lux (max tint), respectively.

As Fig. 23 depicts, indoor spaces will be partially in shadow during the summer solstice, which limits the vertical illuminance at eye level compared to the winter solstice and February 21st, when direct sunlight touches the body.

**Fig. 22:**
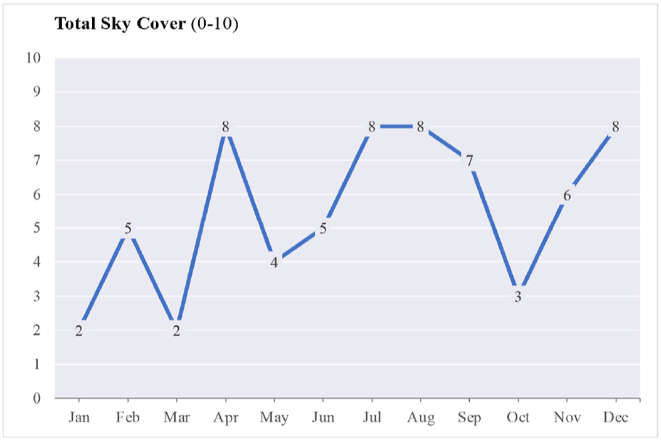
Total sky coverage (scale 0–10, 10: total sky dome coverage) for simulated dates.

The variation in results can also be attributed to the sky and weather conditions, which Lark simulation takes into account by reading the direct normal illuminance (DNI) and the diffuse horizontal illuminance (DHI) from the weather data file to interpret the sky type. The outcomes of the simulation are influenced by the specific sky conditions at the selected time. For instance, as Fig. 22 illustrates, on October 21st (the highest value for vertical illuminance), the sky coverage in the weather file is rated as 3 on a scale of 0–10. Considering that the average annual cloud coverage in Trondheim is 6.3 based on the data from 2004 to 2018, we can expect slightly lower average values for vertical illuminance. This highlights the impact of sky conditions and having the sun in the field of view on the non-visual impacts of daylight indoors.

**Fig. 23:**
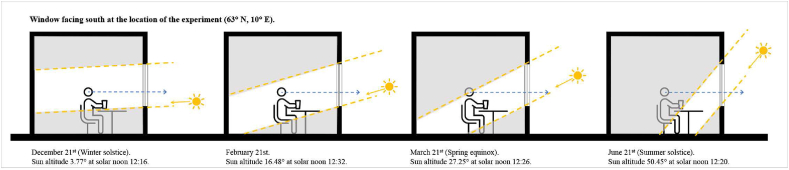
Simplified schematic illustration of the sun's altitude and daylight reaching the eye in an indoor setting.

Secondly, the recommended intensity of light exposure for indoor settings is 250 lux Melanopic EDI during the daytime [49]. However, the Melanopic EDI values (Table 9) in December did not meet the acceptable threshold for the smart glazing (SG_max_: 120 lux and SG_min_: 160 lux at local noon), indicating insufficient light exposure. It is important to note that the data collection sensor was positioned 40 cm close to the glazing surface, looking directly outside (perpendicular to the surface of the glazing), which may not represent typical occupant distances from the glazing in real settings. The reduction in light reaching the eye with an increase in distance from the window suggests that even clear glazing may not meet the required level of the Melanopic EDI throughout December. Although the presented data provides an overview of how different glazing types perform under various sky conditions, further research is needed to consider the distance from the window and the occupant's viewpoint for a comprehensive understanding of the non-visual effects of daylight. Additionally, the duration of light exposure is also important [50,77], especially in the winter when daylight hours are limited, as daylight only lasts 4:43 h on the winter solstice at the location of the experiment. Glare avoidance and visual comfort strategies can also impact the duration of light exposure. While the presence of the sun in the field of view tends to increase the Melanopic EDI values for non-visual effects, this will most likely be mitigated by implementing glare reduction measures like shading devices. Therefore, achieving the right balance between visual comfort and the non-visual impacts of light can be a challenging task that requires careful consideration.

The results also demonstrate that the average Melanopic DER is 0.98 for maximum coloration and 0.77 for minimum coloration (Fig. 15), while Clear glazing has an average of 0.88. This indicates that the smart glazing provides superior Melanopic DER in its maximum coloration despite its lowest transmittance. Moreover, the comparison between average α-opic 10.13039/501100002288DER (Fig. 15) and α-opic EDI (Table 9) indicates that 10.13039/501100002288DER alone cannot assess the transmitted daylight through glazing regarding non-visual metrics, which supports the notion proposed by Cajochen et al. [78] that Melanopic EDI can serve as a reliable predictor of non-visual responses, thereby validating its usefulness in assessing glazing performance.

### 6.2 Visual effects (colour appearance)

A colour appearance analysis method based on CIELAB [69] and IES colour rendition [70] was used for evaluating the transmitted daylight through Clear and Smart glazing. The CIELAB calculations provide convincing results that align with the observations from a previous study [79], while the IES calculation needs to be discussed.

The fidelity index, which compares measured light's appearance to standard samples using normalised data, indicates that the smart glazing provides a good fidelity index (R > 80). However, it was observed that there is a strong hue shift in the purple part of the spectrum, with varying directions (towards yellow or blue). Additionally, the fluctuations in light levels due to weather changes should not be overlooked. While R > 80 is considered good colour rendering, dynamic changes of light level combined with colour shift can be misleading. Previous research has shown that colour rendering with R > 95 compared to R = 80 has various positive effects [80]. An increase in solar radiation leads to a more saturated glazing tint, resulting in lower light transmittance and a shift towards green, while a reduction in solar radiation increases transmission and shifts the colours towards yellow. The human eye adapts well to changes in light level, and it may be perceived as rather stable, but the changes in the colour of light are more noticeable. Therefore, caution should be exercised when applying smart glazing to certain building functions, such as dental clinics, health care centres, or art studios, where accurate colour evaluation is crucial.

Our results indicate that relying solely on the fidelity index is not sufficient and that the level of light should be considered alongside hue and chroma, as proposed by Arbab and Matusiak [81]. Low light levels resulting from the glazing's low transmittance can hinder colour differentiation. Similar difficulties in distinguishing hues for similar (but not equal) colour samples have been observed in previous studies [79], especially in the purple area. This finding should be taken into account in interior colour design, which often follows colour harmony principles. A harmony made by combinations of complementary or split-complementary colours, such as purple and green, may be easily disrupted.

The findings emphasise the importance of the colour of daylight entering the space, as it affects the perception of space, objects, and even mood. Participants in a related experiment [79] with the same smart glazing perceived a colour shift towards yellow in overcast sky conditions, which made the space more enjoyable, which aligns with the findings of Liang et al. [20]. The study also revealed a significant effect of glazing on the appearance of outdoor colours and the outside view. Understanding how new glazing technology manipulates daylight can help create aesthetically pleasing, comfortable, and functional spaces for specific tasks.

In this paper, we discuss colour changes using lightness, chroma, and hue, which have significant roles and a long history in colour communication. These attributes have been carefully studied by Munsell [82], who used them as the three dimensions in the Munsell Colour System [83]. Other terms, such as vividness, depth, and clarity, were later introduced by CIE to demonstrate the relationship between colour and the surrounding environment. While the CIE colour system is the standard in science and technology, many architecture firms communicate using other colour systems such as Munsell, NCS [84], or RAL, which were developed for surface colour classification and practical application. Understanding the terminology of colour change can improve communication about colour.

### 6.3 Limitation and recommended future research

In this study, the *Lark* was evaluated by comparing its spectral irradiance output to measured data. Lark was found to predict values within an acceptable RMSE, MRBE%, and NRMSE%. Despite its limitations, the results demonstrate that the spectral lighting simulation provides a sufficient overview of the spectral power distribution of daylight transmitted through the glazing types. One of the primary hurdles encountered when utilising Lark is the extensive amount and intricacy of input data required to execute the simulation effectively. This particular aspect proves to be highly restrictive and arduous, particularly during the initial stages of the design process, where crucial decisions regarding material surfaces and glazing types are yet to be finalised. Furthermore, the use of Lark demands a high level of proficiency to successfully operate the simulation and accurately interpret the resultant outcomes. It necessitates a comprehensive understanding of the tool's functionalities, parameters, and settings, along with the expertise to discern and analyse the intricate data generated by the simulation.

Another limitation of Lark in this study is the number of channels (9 channels), which may affect the accuracy of the spectrum's portions with short wavelengths (<550 nm) where the α-opic action spectra of S-cone photopsin and melanopsin are located (see figures in Table 11). This is substantial because, while all five photoreceptors might play a role in the non-visual effects of light [48], Melatonin suppression due to long-term (6.5 h) light exposure is primarily driven by melanopsin [50], as S-cones may have a greater impact on alertness [85]. Increasing the number of channels, especially across the blue part of the spectrum, such as B [b1 (380,422), b2 (422,460), b3 (460,498)], can enhance data resolution for analysing these effects.

**Table 11:**
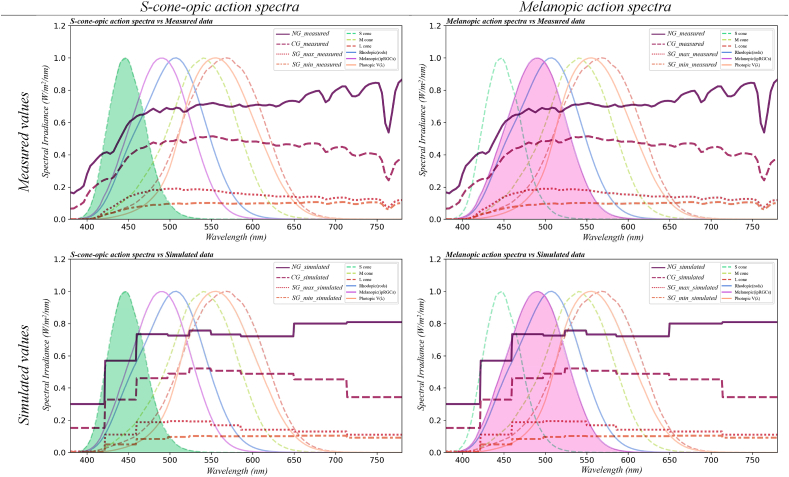
Measured values (top) vs. Simulated values (down) with the spectral sensitivities of the five photoreceptors and the spectral luminous efficiency function for photopic vision (V(λ)) (Highlighted diagrams present S-cone-opic and Melanopic action spectra.).

Furthermore, it is important to acknowledge that the evidence presented in this study is dependent on particular choices made during the simulation configuration, which are bound to certain constraints. These include the SPD values for the sky, the selected dates for simulation, the state of the smart glazing tint, and the level of detail and the materials of the boxes surrounding the measurement points.

First, while employing the D65 spectrum for the sky SPD in our study yielded acceptable error metrics values (see Table 7), enhancing the simulation precision could be achieved by incorporating the measured sky spectrum, which accounts for subtle spectral variances between clear and sunny skies.

Second, since the primary objective of this study was to demonstrate how dynamic variations in sky types and sun altitudes throughout the year affect the studied glazing types, simulated data was exclusively utilised for the 21st day of each month. By taking into account the extreme (max-min) values for sun altitudes and daylight hours, this data serves as a helpful example, providing an overview of changing lighting conditions while capturing vital trends regarding the influence of diverse sky conditions on the investigated glazing types. While it is acknowledged that a higher-resolution approach could yield more detailed insights, we recognise the potential for future research to delve into more comprehensive temporal analyses spanning an entire month rather than just one day. Furthermore, to enhance the annual overview accuracy, it is advisable to automatically adapt the state of smart glazing tint coloration based on the sky condition when it falls between sunny and overcast.

Third, the study's limitations also stem from the simplified representation of indoor elements and surrounding structures. While suitable for the study's aim of comparing glazing types, the use of white boxes in a controlled laboratory setting oversimplified material surfaces, potentially disregarding the influence of complex materials in furniture, walls, ceilings, and floors on the spectral irradiance of light reaching the eye in real-world scenarios. In the simulation model, the surrounding buildings in the area were featured with simplified geometric blocks, and the exterior surface near the window was represented with a plain surface due to the absence of immediate external buildings obstructing sunlight and the lack of greenery or snow during the measurements. Further investigation is required to explore the impact of exterior surfaces (including snow or vegetation), neighbouring buildings, surrounding structures, and interior materials or layout on the non-visual effects of light in the built environment.

Moreover, it is crucial to take the necessary precautions to ensure precise measurement of the spectral reflectance of surfaces in close proximity to the sensor. It was observed that the spectral reflectance of the nearby materials, such as the white boxes adjacent to the glazing types in this study, directly impacts the simulation results’ spectral irradiance. This also highlights the significance of databases for the spectral properties of common materials, which allow designers and researchers to identify the optimal glazing type for their needs, such as the Spectral Materials Database by Jakubiec [86] and IGDB [63].

The findings of this investigation indicate that Lark successfully estimated the spectral irradiance of daylight transmitted through the studied glazing at an acceptable level, consistent with previous studies [[56], [57], [58], [59], [60]]. However, as discussed by Pierson et al. [56], further improvements are necessary before spectral lighting simulation tools can be employed as reliable and efficient decision-making tools for optimising the built environment for occupant non-visual responses in a real-world or field setting.

## 7 Conclusion

This study used a spectral lighting simulation approach for (I) calculating non-visual metrics of transmitted daylight and (II) evaluating the colour appearance of surfaces. To the best of our knowledge, no previous research on electrochromic glazing has examined all α-opic metrics and colour appearance over the course of a year using new spectral lighting simulation techniques. Two glazings, one clear and one electrochromic (ChromoGenics), were tested in sunny and cloudy skies to calibrate and verify a simulation model, resulting in acceptable RSME, MRBE%, and NRMSE% values. This study's novelty is developing a research methodology for a comprehensive evaluation of the performance of glazing in terms of non-visual metrics and colour appearance, taking into account the vast possibilities offered by spectral lighting simulation techniques. The investigation revealed the effect of glazing type on each photoreceptor as well as the variation of the potential non-visual influence of light regarding its possible long-term impact on occupants over the course of the year. The research pursued answers to three research questions.

The first question addressed the potential non-visual effects of smart glazing in different sky conditions throughout the year. Nearly all the α-opic EDI values (S-, M- , L-cone-opic, Rhodopic, and Melanopic) for smart glazing (average of max and min coloration) appear to be about half of the relevant values for clear glazing. This finding indicates that the utilisation of smart glazing, especially in its maximum coloration state, may have a negative impact on occupants concerning non-visual effects. In particular, the limited amount of daylight in dense urban areas or during the winter season emphasises the need for well-considered artificial lighting to support individuals’ productivity and well-being. However, additional research is necessary to determine how occupants' glare avoidance affects nonvisual impacts, particularly during the winter.

The second question assessed the appearance of surface colour and changes caused by the use of smart glazing. In comparison to clear glazing, the hue and chroma values of smart glazing are acceptable for normal-function spaces. However, for certain tasks that demand accurate colour perception, such as in art studios, hospitals, etc., precaution must be considered, as the hue and chroma of purple and blue alter significantly. The colour design of interiors equipped with such glazing should account for the change in appearance of colours (aside from greens) intact with the presence or absence of sunlight and the shift between maximum and minimum coloration of smart glazing. The positive effect of smart glazing is the colour shift towards yellow in the minimum coloration (overcast), which may contribute to a more joyful atmosphere in the room. The negative effect is that in maximum coloration, the vividness of all colours is strongly reduced. Further investigation is required to establish how different types of glazing impact the colour shift of the view out content.

The last question examined the spectral lighting technique's benefits and limitations for the non-visual effects of daylight transmitted through glazing types. Based on the findings, the Lark spectral lighting simulation technique is a promising tool for investigating aspects of light that require SPD evaluation. In this study, the application of this tool provided an estimation of indoor daylight under various sky conditions over the course of the year. The main challenge with using Lark lies in the demanding nature of the input data and the requisite skill set to operate the simulation and interpret the results. On the other hand, the small number of channels in Lark, especially in the short-wave part of the spectrum, may affect the accuracy, for example, in the short-wavelength part of the visible spectrum. Given that our understanding of the non-visual system is an ongoing study area, the accuracy of these spectrum simulation tools should be approached with care. However, minor initiatives performed today are the way to obtain a robust tool in the future.

## Data availability statement

Data will be made available on request.

## Additional information

No additional information is available for this paper.

## CRediT authorship contribution statement

**Marzieh Nazari:** Writing – review & editing, Writing – original draft, Visualization, Validation, Software, Methodology, Investigation, Formal analysis, Data curation, Conceptualization. **Barbara Matusiak:** Writing – review & editing, Supervision, Resources, Project administration, Methodology, Funding acquisition, Conceptualization. **Oliver Stefani:** Writing – review & editing, Validation, Supervision, Conceptualization.

## Declaration of competing interest

The authors declare the following financial interests/personal relationships which may be considered as potential competing interests: Marzieh Nazari reports financial support and equipment, drugs, or supplies were provided by SINTEF.
